# Supplementary material for: Deletion of intestinal Hdac3 remodels the lipidome of enterocytes and protects mice from diet-induced obesity
Source: Nat Commun. 2019 Nov 22;10:5291. doi: 10.1038/s41467-019-13180-8 (PMC6876593; doi:10.1038/s41467-019-13180-8)
Supplement: Supplementary file 1 — Supplementary Information [file 41467_2019_13180_MOESM1_ESM.pdf]

SUPPLEMENTARY INFORMATION

Deletion of intestinal *Hdac3* remodels the lipidome of enterocytes and protects mice from diet-induced obesity.

Dávalos-Salas *et al*.

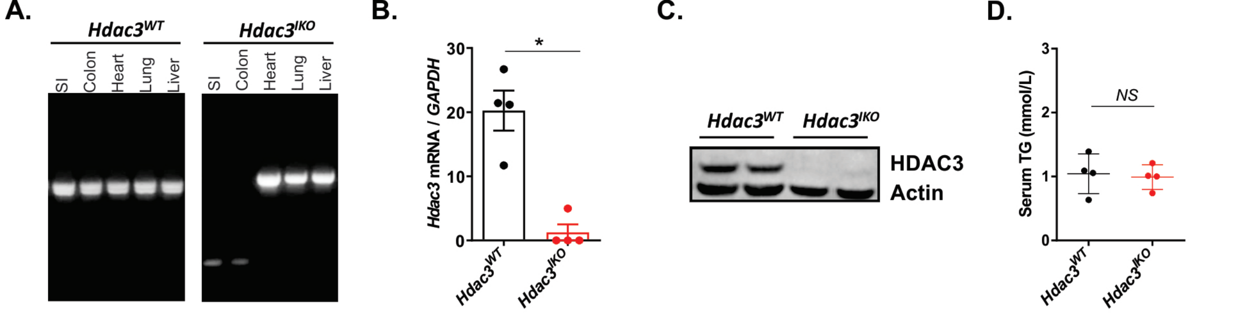

Supplementary Figure 1. Intestinal-specific *Hdac3* deletion confirmed in 6 week old *Hdac3*$^{Lox/Lox}$;*villin*^{Cre} male mice by (**A**) genotyping (SI: small intestine), (**B**) assessment of *Hdac3* mRNA expression by qRT-PCR, and (**C**), assessment of HDAC3 protein expression by western blot, in enterocytes isolated from the entire small intestinal epithelium. Values shown in panel **B** are mean ± SEM of n=4 mice. (**D**) Serum TG levels were determined in blood collected from 4-6 week old male *Hdac3*^{WT} and *Hdac3*^{IKO} mice fed a standard diet and fasted overnight. Values shown are mean±SD, of *n*=4 mice per group. *NS*, not significant, unpaired t test.

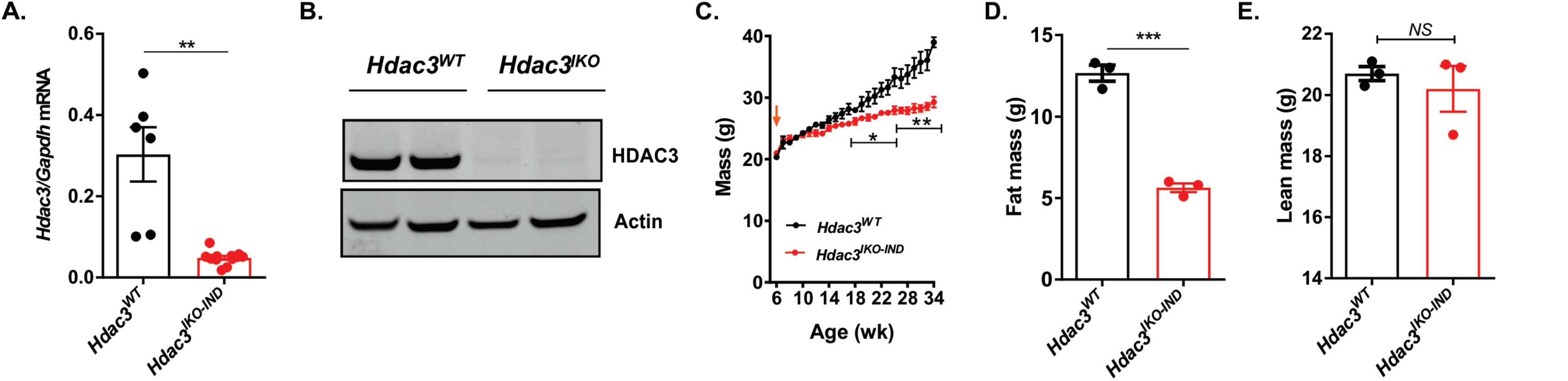

Supplementary Figure 2. (**A-B**) Confirmation of *Hdac3* deletion in the intestinal epithelium in 8 week old *Hdac3-villin*$^{Cre\text{-}ERT2}$ (*Hdac3*$^{IKO\text{-}IND}$) male mice by assessment of (**A**) *Hdac3* mRNA (mean ± SE, n=5 mice) and (**B**) protein expression in IECs isolated from the small intestine. (**C**) Effect of *Hdac3* deletion on body weight. Six-week old *Hdac3-villin*$^{Cre\text{-}ER}$ male mice were injected with vehicle (sunflower oil) or tamoxifen to induce *Hdac3* deletion (orange arrow) and body weight monitored for the following 28 weeks. Values shown are mean ± SEM of n=5 mice per group. (**D**) Fat mass and (**E**) lean mass of *Hdac3*^{WT} and *Hdac3*$^{IND\text{-}IKO}$ mice. Fat and lean mass were determined by DEXA 28 weeks post tamoxifen treatment (34 weeks), in the mice described in panel **C**. Values shown are mean ± SEM, of n = 3 mice, *P<0.05, **P<0.005, ***P<0.0005, *NS*, not significant, unpaired t test.

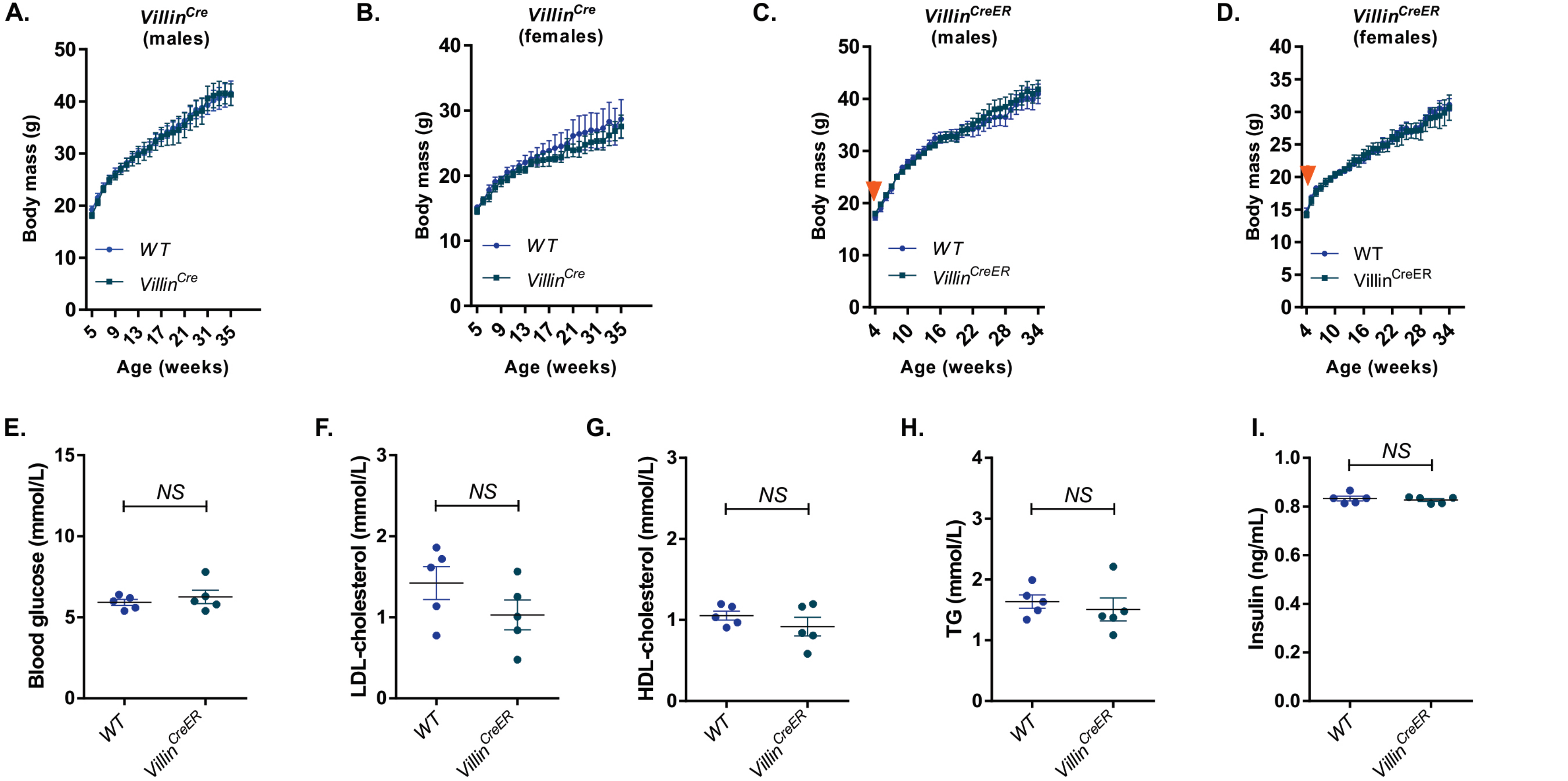

Supplementary Figure 3. Characterization of the metabolic phenotype of *Villin*^{Cre} and *Villin*^{CreER} control mice. (**A, B**) Assessment of body weight of (**A**) male and (**B**) female *Villin*^{Cre} control mice and wild type littermates fed a standard diet over 35 weeks. (**C, D**) Assessment of body weight of (**C**) male and (**D**) female *Villin*^{CreER} mice and wild type littermates fed a standard diet. Mice were treated with tamoxifen at 4 weeks and body weight monitored for the following 30 weeks. (**E-I**) Measurement of (**E**) glucose, (**F**) LDL cholesterol, (**G**) HDL cholesterol, (**H**) triglycerides, and (**I**) insulin in the plasma of 6-8 week old male *Villin*^{CreER} mice and WT littermates 2 weeks post tamoxifen treatment. All mice were fed a standard diet. Blood was collected from mice which were fasted overnight (fasted state) and 2 hours post refeeding. In all cases, values shown are mean ± SEM, of *n*=4 mice per group. *NS*, not significant, unpaired t test.

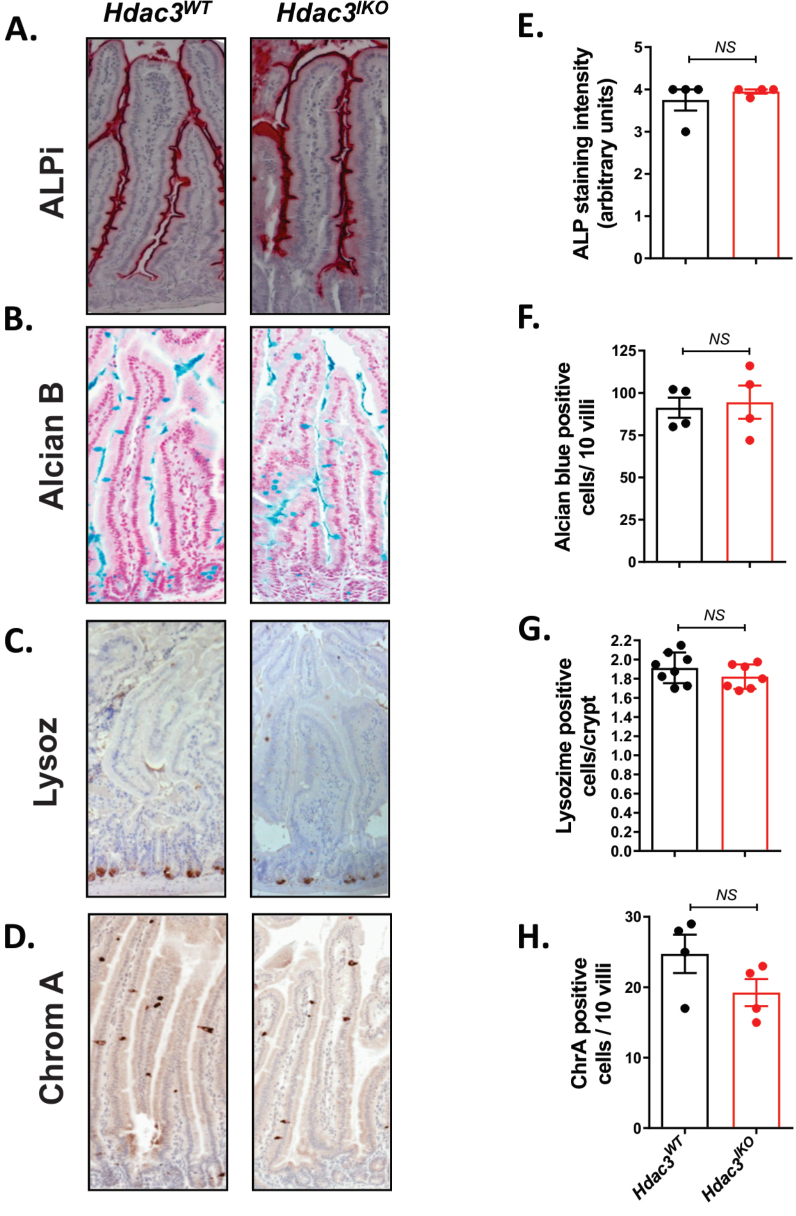

Supplementary Figure 4. Effect of intestinal-specific *Hdac3* deletion on differentiation of the 4 main intestinal cell linages assessed in the duodenum or the duodenum/jejunum border by (**A**) alkaline phosphatase staining for enterocytes, (**B**) alcian blue staining for goblet cells, (**C**) lysozyme staining for Paneth cells and (**D**) Chromogranin A staining for enteroendocrine cells. (**E-H**) Corresponding quantitation of staining intensity or the number of positively stained cells. Values shown are mean ± SEM of *n* =4 male mice per genotype. Mice were 4-6 weeks of age and fed a standard diet. *NS*, not significant, unpaired t test.

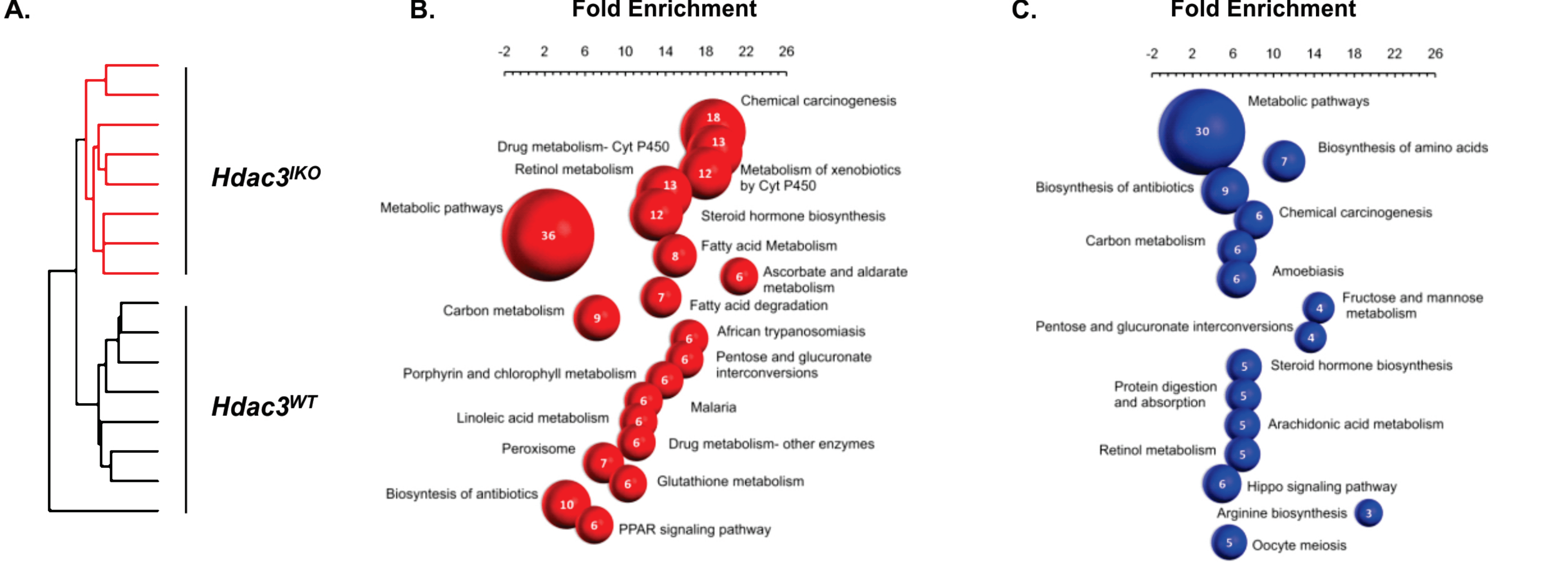

Supplementary Figure 5. (**A**) Unsupervised clustering based on expression of all detected proteins in IECs isolated from 4-6 week old *Hdac3*^{WT} and *Hdac3*^{IKO} male mice fed a standard diet. (**B-C**) Protein set enrichment analysis of proteins (**B**) upregulated or (**C**) downregulated in *Hdac3*^{IKO} mice.

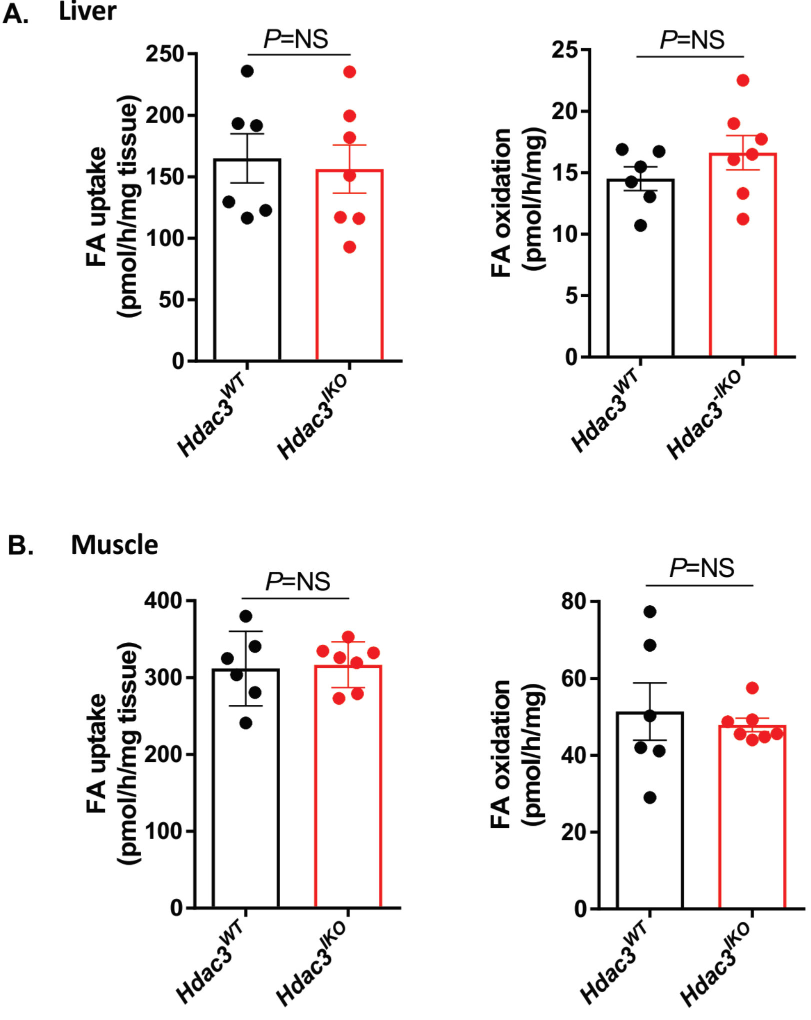

Supplementary Figure 6. Fatty acid uptake (left panels) and oxidation (right panels) in (**A**) liver and (**B**) muscle from 4-6 week old male *Hdac3*^{WT} and *Hdac3*^{IKO} mice fed a standard diet. Values shown are mean ± SEM of *n*=6 and n=7 mice for *Hdac3*^{WT} and *Hdac3*^{IKO} respectively. NS, not significant, unpaired t test.

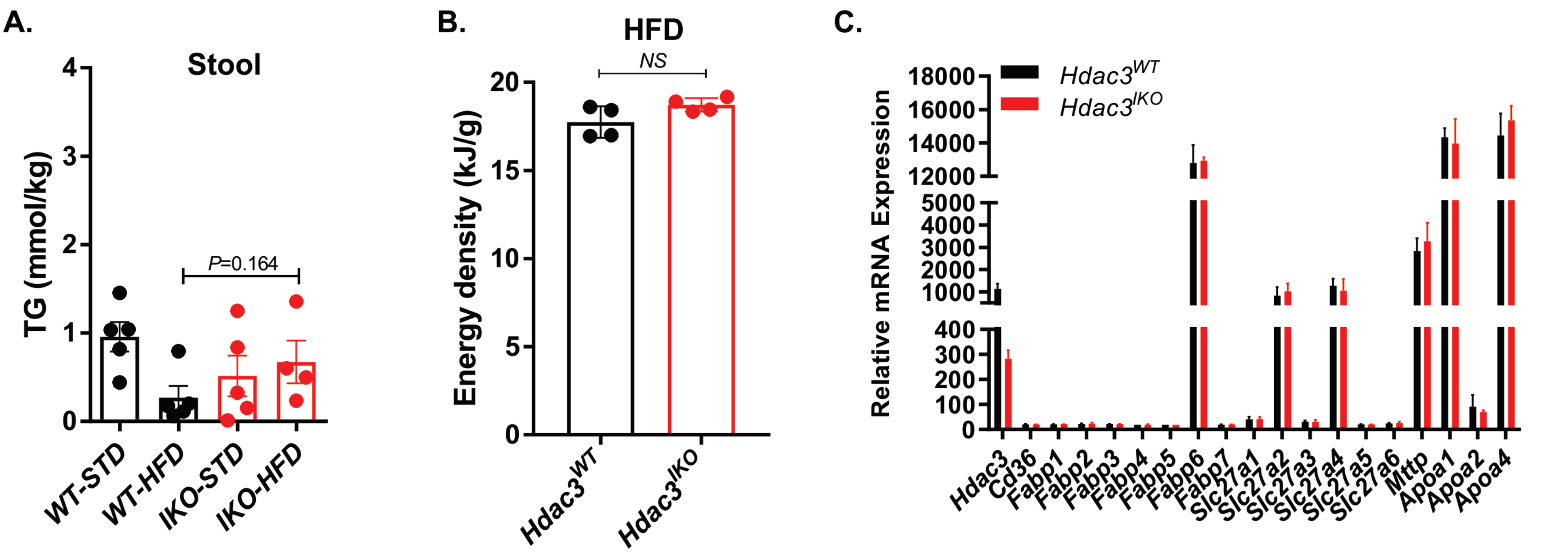

Supplementary Figure 7. (**A**) Triglyceride (TG) levels in stool of 4-6 week old *Hdac3*^{WT} and *Hdac3*^{IKO} male mice fed a STD or HF Diet for 4 weeks. (**B**) Energy density of stool collected from 4-6 week old *Hdac3*^{WT} and *Hdac3*^{IKO} male mice fed a HF Diet assessed by bomb calorimetry. (**C**) Expression of genes involved in lipid uptake and transport in 4-6 week old *Hdac3*^{WT} and *Hdac3*^{IKO} male mice fed a standard diet analysed by microarray. Values shown are mean ± SEM from 3 mice for each genotype. Groups were compared using an unpaired t test.

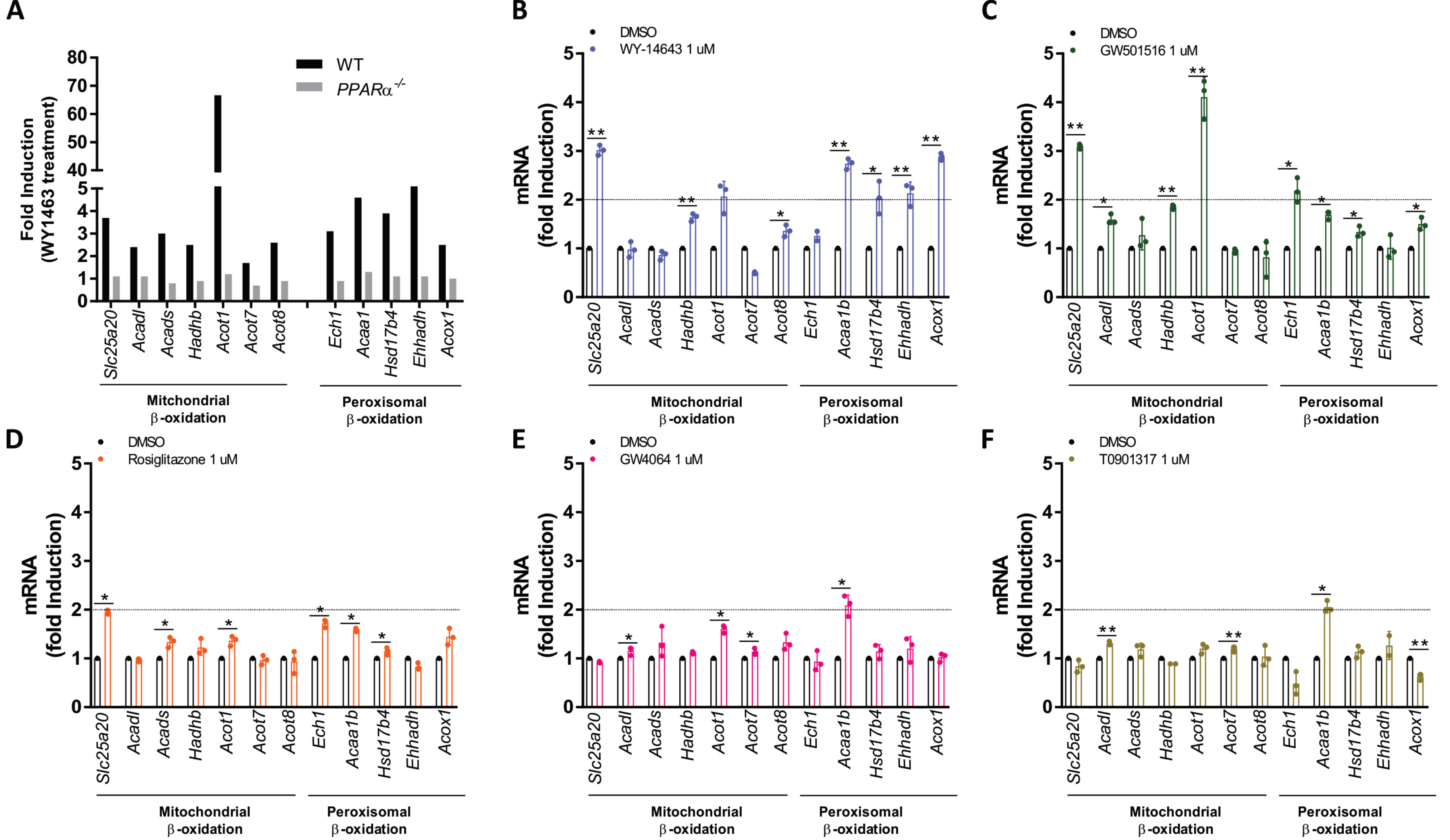

Supplementary Figure 8. (A) Expression of genes involved in mitochondrial and peroxisomal β-oxidation in WT and *PPAR*α$^{-/-}$ mice treated with the PPARα agonist WY14643. Male mice, 4-5 months old, were treated with 0.1% WY14643 for 5 days and gene expression changes in enterocytes assessed by Affymetrix microarray analysis. Data extracted from Bunger *et al,* Physiol. Genomics 30:2, 192-204. **(B-E)** Effect of nuclear receptor agonists on mitochondrial and peroxisomal β-oxidation gene expression in small intestinal organoids generated from WT mice. Organoids derived from the small intestine of a single mouse were expanded into 24 well plates containing ~300 organoids per well and stimulated with agonists of (**B**) PPARα (WY-14643, 1 μM), (**C**) PPARβ/δ (GW501516, 1 μM), (**D**) PPARγ (rosiglitazone, 1 μM), (**E**) FXR (GW4064, 1 μM) or (**F**) LXR (T0901317, 1 μM) for 6h, and gene expression changes determined by qPCR. Values shown are from a representative experiment performed in triplicate. Experiments were performed on 2 separate occasions using organoids derived from independent mice. *P<0.05, **P<0.005, unpaired t test.

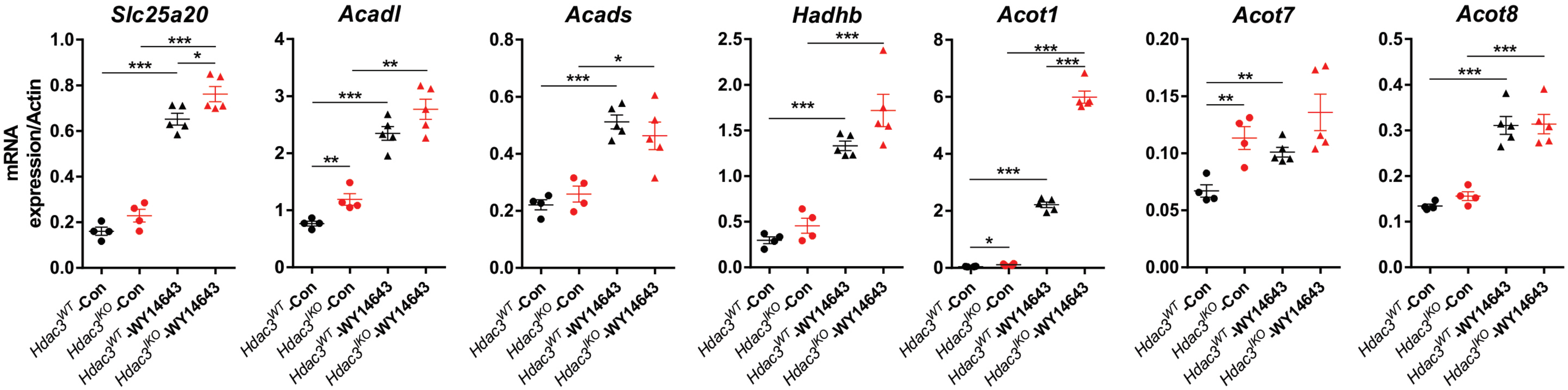

Supplementary Figure 9. *Hdac3^{WT}* and *Hdac3^{IKO}* male mice 4-6 weeks of age were fed a standard chow diet supplemented with or without WY14643 (WY) (0.1% w/w) for 24h and gene expression changes in the duodenum determined by qPCR. Values shown are mean + SEM of n=4 mice per treatment group, **P*<0.05, ***P*<0.005, unpaired t test.

Supplementary Table 1. KEGG pathway analysis of genes upregulated in *Hdac3*^{IKO} mice.

Category	Term	Count	%	P Value	Genes	List Total	Pop Hits	Fold Enrichment	Benjamini	FDR
**KEGG_PATHWAY**	mmu00980:Metabolism of xenobiotics by cytochrome P450	16	0.0233	3.58E-10	GSTA3, EPHX1, GSTT2, CBR3, GSTM6, GSTM5, GSTM1, GSTM2, CBR1, GSTM3, UGT2B36, UGT2B35, GSTM4, ADH4, GSTP2, GSTP1	231	64	8.35	0.0000001	0.0000005
**KEGG_PATHWAY**	mmu05204:Chemical carcinogenesis	18	0.0262	1.23E-09	GSTA3, CYP3A25, CYP3A11, EPHX1, GSTT2, GSTM6, GSTM5, GSTM1, GSTM2, CBR1, GSTM3, UGT2B36, UGT2B35, GSTM4, ADH4, SULT1A1, GSTP2, GSTP1	231	92	6.54	0.0000001	0.0000016
**KEGG_PATHWAY**	mmu00982:Drug metabolism - cytochrome P450	13	0.0190	4.35E-07	GSTA3, GSTT2, GSTM6, GSTM5, GSTM1, GSTM2, GSTM3, UGT2B36, UGT2B35, GSTM4, ADH4, GSTP2, GSTP1	231	66	6.58	0.0000340	0.0005598
**KEGG_PATHWAY**	mmu01100:Metabolic pathways	68	0.0991	9.53E-07	SEPHS2, EHHADH, G6PDX, PIP5K1B, PPCS, ACOT1, HLCS, CKB, ACOT8, NANS, AGPS, NT5C2, PRIM2, MGLL, ATP5O, COX17, HMGCL, ACAA2, HYAL1, DDC, NDUFB11, ACADS, CYP3A11, FBP1, CBR3, ACADL, PMM2, COQ5, NME7, PANK3, CYP4A31, OAT, CYP3A25, NFS1, UROS, ZNRD1, PPT1, ASNS, POLA2, ASL, ALDH3A2, HADHB, CBR1, TPI1, ADH4, DAD1, PNPO, HSD17B6, ENO3, GALE, LIAS, UCK1, RPIA, HSD17B4, UCK2, PTS, ACAA1B, TCIRG1, NDUFA5, TBXAS1, GALT, AK2, AKR1B8, UGT2B36, UGT2B35, PSAT1, GUK1, ALG12	231	1278	1.78	0.0000557	0.0012246
**KEGG_PATHWAY**	mmu00480:Glutathione metabolism	11	0.0160	4.09E-06	GSTM1, GSTM2, GSTA3, GSTM3, GSTM4, G6PDX, GSTT2, GSTM6, GSTM5, GSTP2, GSTP1	231	55	6.68	0.0001912	0.0052524
**KEGG_PATHWAY**	mmu00071:Fatty acid degradation	9	0.0131	8.63E-05	ACAA2, ACADS, EHHADH, ADH4, CYP4A31, ACADL, ACAA1B, ALDH3A2, HADHB	231	49	6.14	0.0033585	0.1108319
**KEGG_PATHWAY**	mmu01212:Fatty acid metabolism	9	0.0131	1.16E-04	SCD1, ACAA2, SCD2, ACADS, EHHADH, PPT1, ACADL, ACAA1B, HADHB	231	51	5.90	0.0038604	0.1486378
**KEGG_PATHWAY**	mmu04146:Peroxisome	11	0.0160	1.64E-04	ECH1, ACOT8, NUDT12, AGPS, EHHADH, PXMP4, MPV17, CRAT, HSD17B4, ACAA1B, HMGCL	231	83	4.43	0.0047779	0.2102774
**KEGG_PATHWAY**	mmu04141:Protein processing in endoplasmic reticulum	14	0.0204	0.001475243	RAD23B, WFS1, DNAJC10, CALR, UBE2D3, ATF4, TXNDC5, BAX, BAG2, DAD1, NFE2L2, MBTPS1, SEC61G, SSR3	231	168	2.78	0.0376573	1.8800317
**KEGG_PATHWAY**	mmu03050:Proteasome	7	0.0102	0.00201688	PSMB4, PSMB6, PSMD12, PSME2, PSMD11, POMP, PSMD7	231	45	5.20	0.0461441	2.5620670
**KEGG_PATHWAY**	mmu01130:Biosynthesis of antibiotics	15	0.0219	0.004692959	ACAA2, EHHADH, G6PDX, FBP1, AK2, ASL, ALDH3A2, NME7, HADHB, TPI1, ENO3, RPIA, PSAT1, OAT, ACAA1B	231	214	2.34	0.0952232	5.8681099
**KEGG_PATHWAY**	mmu00280:Valine, leucine and isoleucine degradation	7	0.0102	0.005609112	ACAA2, ACADS, EHHADH, ACAA1B, ALDH3A2, HMGCL, HADHB	231	55	4.25	0.1038842	6.9759447
**KEGG_PATHWAY**	mmu00062:Fatty acid elongation	5	0.0073	0.006850616	ACAA2, ACOT7, ACOT1, PPT1, HADHB	231	26	6.43	0.1163864	8.4580216
**KEGG_PATHWAY**	mmu01040:Biosynthesis of unsaturated fatty acids	5	0.0073	0.007857538	SCD1, ACOT7, SCD2, ACOT1, ACAA1B	231	27	6.19	0.1235292	9.6440338
**KEGG_PATHWAY**	mmu03320:PPAR signaling pathway	8	0.0117	0.009548205	SCD1, PPARA, SCD2, EHHADH, CYP4A31, ACADL, DBI, ACAA1B	231	80	3.34	0.1390059	11.6035750
**KEGG_PATHWAY**	mmu00240:Pyrimidine metabolism	8	0.0117	0.033696233	PRIM2, NT5C2, ENTPD5, ZNRD1, UCK1, UCK2, POLA2, NME7	231	103	2.60	0.3942583	35.6385547

**KEGG_PATHWAY**	mmu00830:Retinol metabolism	7	0.0102	0.049159537	UGT2B36, UGT2B35, CYP3A25, CYP3A11, ADH4, CYP4A31, HSD17B6	231	89	2.63	0.5003587	47.6931576
**KEGG_PATHWAY**	mmu01200:Carbon metabolism	8	0.0117	0.057660565	TPI1, ACADS, EHHADH, G6PDX, FBP1, ENO3, RPIA, PSAT1	231	116	2.30	0.5379428	53.3966060
**KEGG_PATHWAY**	mmu00030:Pentose phosphate pathway	4	0.0058	0.058722311	G6PDX, FBP1, RBKS, RPIA	231	30	4.46	0.5254155	54.0671515
**KEGG_PATHWAY**	mmu00051:Fructose and mannose metabolism	4	0.0058	0.07947037	AKR1B8, TPI1, FBP1, PMM2	231	34	3.93	0.6204736	65.5110266
**KEGG_PATHWAY**	mmu00980:Metabolism of xenobiotics by cytochrome P450	16	0.0233	3.58E-10	GSTA3, EPHX1, GSTT2, CBR3, GSTM6, GSTM5, GSTM1, GSTM2, CBR1, GSTM3, UGT2B36, UGT2B35, GSTM4, ADH4, GSTP2, GSTP1	231	64	8.35	0.0000001	0.0000005
**KEGG_PATHWAY**	mmu05204:Chemical carcinogenesis	18	0.0262	1.23E-09	GSTA3, CYP3A25, CYP3A11, EPHX1, GSTT2, GSTM6, GSTM5, GSTM1, GSTM2, CBR1, GSTM3, UGT2B36, UGT2B35, GSTM4, ADH4, SULT1A1, GSTP2, GSTP1	231	92	6.54	0.0000001	0.0000016
**KEGG_PATHWAY**	mmu00982:Drug metabolism - cytochrome P450	13	0.0190	4.35E-07	GSTA3, GSTT2, GSTM6, GSTM5, GSTM1, GSTM2, GSTM3, UGT2B36, UGT2B35, GSTM4, ADH4, GSTP2, GSTP1	231	66	6.58	0.0000340	0.0005598

Supplementary Table 2. Proteins differentially expressed in IECs of *Hdac3*^{IKO} and *Hdac3*^{WT} mice. Values shown are the Average and SD of n=8 independent mice per group. Groups were compared using an unpaired Student's t test, with *P*<0.05 considered statistically significant.

Gene Symbol	IKO Ave	IKO SD	WT Ave	WT SD	IKO/WT	T Test
Cyp3a11	24.780	11.402	6.242	1.796	3.970	0.0005
Ces1f	18.572	7.516	5.746	1.467	3.232	0.0003
Gstm1	36.510	22.610	12.828	7.901	2.846	0.0143
Por	20.182	8.019	7.840	3.115	2.574	0.0012
Cyp3a41a	12.980	6.154	5.230	0.333	2.482	0.0032
Cyp3a41b	12.980	6.154	5.230	0.333	2.482	0.0032
Shmt2	13.616	5.317	5.726	1.330	2.378	0.0011
Gstm3	37.515	8.955	16.974	10.855	2.210	0.0010
Ehhadh	69.790	7.340	32.888	9.307	2.122	0.0000
Cyp3a44	13.005	5.748	6.292	2.043	2.067	0.0076
Clca4b	13.257	6.680	6.744	2.952	1.966	0.0244
Tstd1	10.240	1.069	5.230	0.333	1.958	0.0000
Car2	10.859	2.240	5.726	1.330	1.896	0.0001
Fabp1	31.389	11.711	16.620	2.174	1.889	0.0035
Gstm2	15.442	7.843	8.271	3.444	1.867	0.0328
Gstm7	15.442	7.843	8.271	3.444	1.867	0.0328
Gstm4	14.322	7.526	7.774	2.929	1.842	0.0378
Hadhb	54.155	12.884	29.514	9.456	1.835	0.0007
Hbb-b2	30.481	14.804	16.804	5.225	1.814	0.0273
Ech1	21.723	4.967	11.990	2.945	1.812	0.0003
Maoa	57.071	18.288	31.668	10.072	1.802	0.0040
Dhrs4	20.572	3.337	11.477	2.125	1.792	0.0000
Lgals4	22.605	11.416	12.651	5.502	1.787	0.0433
Ckb	55.342	5.836	31.367	5.892	1.764	0.0000
Cyp3a16	9.218	2.925	5.230	0.333	1.762	0.0018
Crot	11.882	5.094	6.768	2.071	1.756	0.0198
Hbb-b1	77.082	21.548	44.290	12.963	1.740	0.0024
Gls	18.396	6.736	10.573	3.679	1.740	0.0120
Hbb-bt	100.317	25.633	57.848	15.574	1.734	0.0013
Hbb-bs	100.317	25.633	57.848	15.574	1.734	0.0013
Acadl	10.888	3.391	6.323	2.197	1.722	0.0065
Prdx6	30.796	8.771	18.108	5.477	1.701	0.0037
Cpt1a	8.659	3.984	5.230	0.333	1.656	0.0294
Acad11	8.632	3.774	5.230	0.333	1.650	0.0236
Caprin1	8.595	1.784	5.230	0.333	1.643	0.0001
LOC102642619	29.727	8.630	18.108	5.477	1.642	0.0062
Hba-a2	74.215	19.835	45.814	11.315	1.620	0.0034
Hba-a1	74.215	19.835	45.814	11.315	1.620	0.0034
Cyp3a25	8.467	3.790	5.230	0.333	1.619	0.0305
Clca4a	11.731	4.684	7.290	3.081	1.609	0.0418
Hsd17b11	26.557	4.998	16.696	4.644	1.591	0.0011
Snd1	8.014	2.893	5.230	0.333	1.532	0.0171
B2m	8.750	2.343	5.750	1.490	1.522	0.0085
Gfpt1	21.928	8.291	14.517	4.821	1.510	0.0463
Rpl27a	10.202	2.378	6.768	2.071	1.507	0.0082
Atad3a	8.718	2.242	5.787	1.737	1.506	0.0111
Ddx5	13.906	3.272	9.354	3.042	1.487	0.0121
Acox1	79.807	11.406	53.991	7.430	1.478	0.0001
Hsd17b4	47.065	11.371	31.997	6.840	1.471	0.0063
Gzma	7.642	2.569	5.230	0.333	1.461	0.0197
Mrpl10	7.614	2.480	5.230	0.333	1.456	0.0174
Shmt1	7.608	2.473	5.230	0.333	1.455	0.0174
Sec22b	7.600	2.403	5.230	0.333	1.453	0.0153
4931406C07Rik	22.747	5.291	15.672	3.867	1.451	0.0086
Cpt2	30.998	6.897	21.460	6.301	1.444	0.0119
Anxa13	35.763	6.508	24.972	11.103	1.432	0.0326
Oat	134.294	23.234	94.221	16.068	1.425	0.0013
2210407C18Rik	9.755	0.632	6.859	2.451	1.422	0.0060
Atp5k	19.623	4.497	13.813	5.573	1.421	0.0377
Hspa9	56.847	8.608	40.142	12.733	1.416	0.0082
Acaca	8.194	2.521	5.787	1.737	1.416	0.0431
Hdhd3	10.389	3.149	7.345	2.370	1.414	0.0464
Gstm5	8.125	2.315	5.746	1.467	1.414	0.0278
Npm1	14.604	2.730	10.416	2.948	1.402	0.0106
Ugt2b5	8.753	2.352	6.245	1.813	1.402	0.0316
Ugt2b1	8.753	2.352	6.245	1.813	1.402	0.0316
Ugt2b36	8.753	2.352	6.245	1.813	1.402	0.0316
Ugt2b35	8.753	2.352	6.245	1.813	1.402	0.0316
Ugt2b38	8.753	2.352	6.245	1.813	1.402	0.0316
Ugt2b37	8.753	2.352	6.245	1.813	1.402	0.0316
Me1	8.644	2.009	6.227	1.724	1.388	0.0217
Agr2	27.045	4.889	19.582	6.273	1.381	0.0189
LOC102642602	11.371	3.294	8.365	1.994	1.359	0.0444
Rpl22	11.371	3.294	8.365	1.994	1.359	0.0444
Gpd1	43.914	4.156	32.383	7.161	1.356	0.0015
Coq6	7.034	2.235	5.230	0.333	1.345	0.0405
Rdx	14.104	2.600	10.533	3.441	1.339	0.0345
Msn	14.104	2.600	10.533	3.441	1.339	0.0345
Abhd6	6.980	2.026	5.230	0.333	1.335	0.0303
Lypla1	9.755	0.632	7.349	2.368	1.327	0.0148
Gpd2	47.252	6.861	35.618	9.424	1.327	0.0136
Vars	6.938	1.825	5.230	0.333	1.327	0.0208
Aldob	75.495	10.347	58.630	11.614	1.288	0.0084
Cat	56.470	7.472	44.417	10.258	1.271	0.0177
Cps1	210.807	27.593	166.599	23.485	1.265	0.0039
Hadha	110.866	8.351	87.891	10.291	1.261	0.0002
P4hb	145.066	16.552	116.307	22.351	1.247	0.0111
Ddx17	10.272	1.410	8.326	1.837	1.234	0.0323
Sis	157.392	18.615	128.412	15.522	1.226	0.0045
Tmsb4x	14.096	0.914	11.528	2.435	1.223	0.0144
Eef2	88.627	16.552	73.102	10.067	1.212	0.0398
Hspd1	145.825	16.509	120.757	18.004	1.208	0.0116
Mttp	177.228	19.130	150.445	21.301	1.178	0.0192

Mdh2	90.537	13.927	77.058	8.569	1.175	0.0352
Lct	12.256	4.627	41.762	13.513	0.293	0.0000
Asah2	6.454	1.822	21.482	7.672	0.300	0.0001
Serpinb6a	7.909	3.853	21.138	8.794	0.374	0.0016
Iqgap2	5.977	1.689	14.819	8.136	0.403	0.0094
Lta4h	7.938	4.612	19.617	12.997	0.405	0.0311
Mpp1	9.638	1.799	22.446	4.133	0.429	0.0000
Serpinb1a	13.180	7.783	30.661	11.146	0.430	0.0027
Cyp2c65	9.990	5.452	22.026	4.774	0.454	0.0003
Vat1	8.539	3.416	18.721	4.880	0.456	0.0003
Ace	12.246	5.412	26.730	8.899	0.458	0.0015
Khk	7.559	2.268	16.180	2.935	0.467	0.0000
Aadac	5.937	1.420	12.441	3.221	0.477	0.0001
Ephx2	18.895	5.550	39.109	8.300	0.483	0.0001
Mme	9.638	1.799	19.356	5.613	0.498	0.0004
Dnpep	5.417	0.351	10.854	4.608	0.499	0.0050
Epb4.1l3	5.417	0.351	10.843	3.336	0.500	0.0004
Aco1	9.565	3.484	19.129	7.049	0.500	0.0040
Sult1d1	7.048	2.297	14.069	2.337	0.501	0.0000
Dpp4	5.417	0.351	10.727	6.418	0.505	0.0348
Cltc	14.951	5.125	29.533	10.953	0.506	0.0042
Cyp2c66	8.502	3.336	16.754	3.917	0.507	0.0005
Naaladl1	12.002	6.367	23.255	6.812	0.516	0.0042
Glud1	31.535	8.354	60.996	10.460	0.517	0.0000
Slc15a1	7.072	2.389	13.146	3.808	0.538	0.0019
Sord	5.902	1.171	10.891	3.631	0.542	0.0024
Ywhah	9.072	3.246	16.447	5.976	0.552	0.0084
Cndp2	33.271	10.970	60.183	11.527	0.553	0.0003
Tspan8	8.499	3.320	15.277	4.885	0.556	0.0059
Arf5	11.071	6.369	19.424	8.367	0.570	0.0413
Pdzk1	5.969	1.638	10.470	2.899	0.570	0.0019
Fam213a	7.095	3.446	12.401	4.879	0.572	0.0249
Tgm2	5.933	1.397	10.296	3.969	0.576	0.0109
Rmdn3	5.417	0.351	9.384	2.955	0.577	0.0021
Ugt2a3	5.417	0.351	9.358	2.029	0.579	0.0001
Sfn	8.555	2.744	14.535	3.977	0.589	0.0035
Dhrs11	7.081	3.383	12.030	3.804	0.589	0.0157
Clic5	8.050	2.012	13.485	2.663	0.597	0.0004
Acy1	6.577	2.402	10.903	4.185	0.603	0.0238
Psap	8.652	3.124	14.127	3.639	0.612	0.0061
Rab7	6.418	1.621	10.461	2.012	0.614	0.0006
Nt5c	5.417	0.351	8.825	4.472	0.614	0.0497
Myo1a	9.278	3.050	15.030	3.149	0.617	0.0023
Copb1	8.613	2.820	13.899	4.494	0.620	0.0137
Xdh	10.738	4.359	17.195	4.567	0.624	0.0118
Abat	5.977	1.689	9.426	3.231	0.634	0.0181
Cth	6.578	2.407	10.359	4.000	0.635	0.0380
Ap2a2	5.962	1.592	9.331	2.847	0.639	0.0112
Cmpk1	10.750	2.874	16.552	5.501	0.649	0.0193
Picalm	6.454	1.822	9.892	2.458	0.652	0.0067
Cyp2b10	13.413	6.635	20.347	2.687	0.659	0.0160
Txnrd1	7.983	2.713	11.913	4.178	0.670	0.0425
Mep1b	23.067	6.068	34.362	8.766	0.671	0.0096
Casp1	5.902	1.171	8.777	2.276	0.672	0.0067
Ppp2r1a	8.128	2.325	11.921	2.576	0.682	0.0080
Tax1bp3	5.417	0.351	7.880	2.345	0.687	0.0108
Mgam	97.024	28.915	140.902	25.079	0.689	0.0059
Myo1b	6.529	2.169	9.418	0.600	0.693	0.0027
Npc1l1	5.417	0.351	7.781	2.870	0.696	0.0365
Ptgr1	6.496	2.020	9.331	2.847	0.696	0.0376
Pfkl	5.417	0.351	7.776	1.959	0.697	0.0047
Eps8l2	5.417	0.351	7.751	2.773	0.699	0.0332
Gm11226	14.885	5.549	21.171	4.969	0.703	0.0317
Ywhaq	14.885	5.549	21.171	4.969	0.703	0.0317
Ilvbl	6.938	1.825	9.820	2.222	0.707	0.0133
Myo7b	11.804	4.392	16.654	4.482	0.709	0.0463
Psme1	15.179	3.921	21.405	4.798	0.709	0.0131
Tagln2	17.897	3.714	25.098	6.519	0.713	0.0168
Wdr1	9.722	3.180	13.545	1.913	0.718	0.0113
Txndc17	7.529	2.155	10.484	2.163	0.718	0.0160
Pkm	64.354	9.716	89.304	12.805	0.721	0.0006
Fahd1	11.371	3.294	15.667	2.139	0.726	0.0079
Pgam1	11.384	2.354	15.634	3.701	0.728	0.0159
Cryl1	6.447	1.783	8.842	1.328	0.729	0.0087
Eci1	14.537	4.885	19.933	4.884	0.729	0.0443
Gsto1	12.467	2.407	17.038	4.328	0.732	0.0206
Copg1	12.482	3.363	17.039	4.389	0.733	0.0352
Slc3a2	22.643	6.773	30.844	7.669	0.734	0.0398
Pepd	19.582	6.433	26.660	6.496	0.735	0.0460
Fbp2	22.231	1.956	30.152	9.572	0.737	0.0378
Gna11	16.133	5.008	21.855	5.070	0.738	0.0395
Xpnpep1	20.488	4.216	27.655	6.735	0.741	0.0231
Cdhr5	5.417	0.351	7.310	2.241	0.741	0.0333
Gnaq	5.417	0.351	7.308	2.211	0.741	0.0315
Aldh1l1	5.417	0.351	7.284	2.125	0.744	0.0280
Hsd17b10	13.603	4.109	18.272	3.357	0.744	0.0260
Ctnna1	32.947	12.709	44.167	7.231	0.746	0.0477
Anxa2	48.604	12.867	64.338	8.644	0.755	0.0123
Prdx5	19.421	4.592	25.449	6.142	0.763	0.0432
Ywhag	10.275	1.443	13.451	3.167	0.764	0.0218
Rab1	16.023	4.344	20.947	3.809	0.765	0.0302
Arg2	32.001	3.772	41.765	11.231	0.766	0.0352
Lap3	29.679	6.808	38.731	9.641	0.766	0.0478
Dak	45.272	11.860	58.889	10.780	0.769	0.0307
Ugt1a1	29.175	6.639	35.954	3.229	0.811	0.0211
Prkar2a	12.439	2.262	15.197	2.491	0.819	0.0361
Ywhaz	33.401	5.740	40.524	7.300	0.824	0.0477

Supplementary Table 3. List of primers used in quantitative real time PCR

Name	Sequence 5'-3'
Hdac3 F	GCATTCGAGGACATGGGGAA
Hdac3 R	TTTCGGACAGTGTAGCCACC
Gapdh F	CGACCCCTTCATTGACCTTA
Gapdh R	GCCTTGACTGTGCTGTTGAA
Slc25a20 F	AGCAGAAATCTCCAGAGGATGA
Slc25a20 R	GCATTTGATCCGTTCTCCAG
Acadl F	GCCAAAAGATCTGGGAGTGA
Acadl R	ATCGAGCTTCACGGTTGGT
Acads F	CATCTCTTCCCCACAGCTCA
Acads R	CAGGTAATCCAAGCCTGCAC
Hadhb F	TCCAGCTCACACTGTCACCA
Hadhb R	TAACTCAACACCACCAGCCA
Acot1 F	TACGATGACCTCCCCAAGAA
Acot1 R	AGCCCAATTCCAGGTCCTTT
Acot7 F	TACAGCCAGTCCAGCCTGAT
Acot7 R	ACAATCCCAGCCACCTCAT
Acot8 F	TCCACTCCCTGCACTGCTAC
Acot8 R	GGCTCCTGTCCGTATCCTCT
Ech1 F	GGAGGGAGTTGGTGGAAT
Ech1 R	CACAGGCAGAAACGAGGT
Acaa1b F	ACTTCGGAGAATGTGGCTGA
Acaa1b R	CTCAGCATGGAAGCATCCTC
Hsd17b4 F	TGCAATACTCTCGCCATTGA
Hsd17b4 R	GCTTCAGGGCTTCAACAAGA
Ehhadh F2	CCCAGTTGAAGAAGCCATCA
Ehhadh R2	TGGGACTGGCTTGTTTAGGA
Acox1 F	CAGGAAGAGCAAGGAAGTGG
Acox1 R	CCTTTCTGGCTGATCCCATA

Supplementary Table 4. ChIP primers and location of putative PPREs in fatty acid oxidation genes

Primer	Seq 5'-3'	Product	Putative PPRE	% Match to consensus PPRE	Location
ACOX1 prom F1	GGCCCTGGCCAATCGGTTG	166	**AGGTAAAAGGTCA**	87	minus strand +51
ACOX1 prom R1	GTCCCCGAGCGGCTCCTC				
ACOT8 prom F	CACTCCTTGAAGTCCTCGTAG	182	**GGGTCAGAGGTCA**	74	plus strand -356
ACOT8 prom R	CCTCCTGTTCTGGGCTCC				
ACAA1B prom F4	CCCTGGGGTCTGAGAAAACAC	131	**AGGCCAGAGGTCA**	73	minus strand -955
ACAA1B prom R4	GTGAATGGACCGGGGCTTCC				
ACOT1 prom F2	GCAGTTGCTTCATTCGCAGG	158	**GGGAGAAAGGTCA**	57	plus strand -1041
ACOT1 prom R2	AGTGTAGAATGCAGGCGTTAGG				
