# Supplementary material for: Deletion of intestinal Hdac3 remodels the lipidome of enterocytes and protects mice from diet-induced obesity
Source: Nat Commun. 2019 Nov 22;10:5291. doi: 10.1038/s41467-019-13180-8 (PMC6876593; doi:10.1038/s41467-019-13180-8)
Supplement: Supplementary file 3 — Description of Additional Supplementary Files [file 41467_2019_13180_MOESM3_ESM.pdf]

Description of Additional Supplementary Files

File name: Supplementary Data 1

Description: Genes differentially expressed in IECs of *Hdac3*^{IKO} mice.
