# Supplementary material for: Deletion of intestinal Hdac3 remodels the lipidome of enterocytes and protects mice from diet-induced obesity
Source: Nat Commun. 2019 Nov 22;10:5291. doi: 10.1038/s41467-019-13180-8 (PMC6876593; doi:10.1038/s41467-019-13180-8)
Supplement: Supplementary file 5 — Reporting Summary [file 41467_2019_13180_MOESM5_ESM.pdf]

nature research

Corresponding author(s): John Mariadason

Last updated by author(s): Aug 19, 2019

# Reporting Summary

Nature Research wishes to improve the reproducibility of the work that we publish. This form provides structure for consistency and transparency in reporting. For further information on Nature Research policies, see Authors & Referees and the Editorial Policy Checklist.

## Statistics

For all statistical analyses, confirm that the following items are present in the figure legend, table legend, main text, or Methods section.

| n/a | Confirmed | |
|---|---|---|
| ☐ | ☒ | The exact sample size (*n*) for each experimental group/condition, given as a discrete number and unit of measurement |
| ☐ | ☒ | A statement on whether measurements were taken from distinct samples or whether the same sample was measured repeatedly |
| ☐ | ☒ | The statistical test(s) used AND whether they are one- or two-sided<br>*Only common tests should be described solely by name; describe more complex techniques in the Methods section.* |
| ☒ | ☐ | A description of all covariates tested |
| ☐ | ☒ | A description of any assumptions or corrections, such as tests of normality and adjustment for multiple comparisons |
| ☐ | ☒ | A full description of the statistical parameters including central tendency (e.g. means) or other basic estimates (e.g. regression coefficient) AND variation (e.g. standard deviation) or associated estimates of uncertainty (e.g. confidence intervals) |
| ☐ | ☒ | For null hypothesis testing, the test statistic (e.g. *F*, *t*, *r*) with confidence intervals, effect sizes, degrees of freedom and *P* value noted<br>*Give P values as exact values whenever suitable.* |
| ☒ | ☐ | For Bayesian analysis, information on the choice of priors and Markov chain Monte Carlo settings |
| ☒ | ☐ | For hierarchical and complex designs, identification of the appropriate level for tests and full reporting of outcomes |
| ☒ | ☐ | Estimates of effect sizes (e.g. Cohen's *d*, Pearson's *r*), indicating how they were calculated |

*Our web collection on statistics for biologists contains articles on many of the points above.*

## Software and code

Policy information about availability of computer code

**Data collection**

1) ViiA 7 Real-Time PCR System + Application software version1.2 (Applied Biosystems) - for qPCR data acquisition
2) Aperio ImageScope v11.2.0.780 software - used for image acquisition of immunhistochemically stained sections
3) Odyssey infrared imaging system + Application software version 3.0 (LI-COR Biosciences) - used to image immunoblotted membranes
4) Body composition of mice were analysed using the PIXImus software (version 2.10)
5) Illumina microarray data was normalized using bioconductor
6) For lipid profiling, data was processed using MultiQuant 2.1 software.
7) PPAR response elements (PPREs) were identified in gene promoters using the Dragon PPRE spotter tool (V2.0), using the default threshold of 50%.

**Data analysis**

1) Microsoft Excel and Prism 7.0c (GraphPad Software, Inc.) - used to generated graphs and perform statistics

For manuscripts utilizing custom algorithms or software that are central to the research but not yet described in published literature, software must be made available to editors/reviewers. We strongly encourage code deposition in a community repository (e.g. GitHub). See the Nature Research guidelines for submitting code & software for further information.

## Data

Policy information about availability of data

All manuscripts must include a data availability statement. This statement should provide the following information, where applicable:

- Accession codes, unique identifiers, or web links for publicly available datasets
- A list of figures that have associated raw data
- A description of any restrictions on data availability

All relevant data are available from the corresponding author upon reasonable request.

# Field-specific reporting

Please select the one below that is the best fit for your research. If you are not sure, read the appropriate sections before making your selection.

☒ Life sciences ☐ Behavioural & social sciences ☐ Ecological, evolutionary & environmental sciences

For a reference copy of the document with all sections, see nature.com/documents/nr-reporting-summary-flat.pdf

# Life sciences study design

All studies must disclose on these points even when the disclosure is negative.

| | |
|---|---|
| Sample size | Sample sizes are provided in the figure legends |
| Data exclusions | In some cases samples or mice which were clear outliers were excluded. |
| Replication | All data presented has been replicated. Most graphs display the collective data from several independent experiments and the number of replicate experiments included are given in the figure legends. |
| Randomization | Genetic mouse experiment: Different genotypes from the same litter were compared. |
| Blinding | During assessment of body weights and body composition assessor did not know the genotypes or treatments the mice received. |

# Reporting for specific materials, systems and methods

We require information from authors about some types of materials, experimental systems and methods used in many studies. Here, indicate whether each material, system or method listed is relevant to your study. If you are not sure if a list item applies to your research, read the appropriate section before selecting a response.

## Materials & experimental systems

| n/a | Involved in the study | |
|---|---|---|
| ☐ | ☒ | Antibodies |
| ☒ | ☐ | Eukaryotic cell lines |
| ☒ | ☐ | Palaeontology |
| ☐ | ☒ | Animals and other organisms |
| ☒ | ☐ | Human research participants |
| ☒ | ☐ | Clinical data |

## Methods

| n/a | Involved in the study | |
|---|---|---|
| ☒ | ☐ | ChIP-seq |
| ☒ | ☐ | Flow cytometry |
| ☒ | ☐ | MRI-based neuroimaging |

## Antibodies

| | |
|---|---|
| Antibodies used | 1) WB antibodies: Used primary antibodies were goat anti-mouse IL-33 (R&D Systems, Cat# AF3626, RRID:AB_884269) and rabbit anti-mouse Gapdh (Sigma-Aldrich, Cat# G9545, RRID:AB_796208). Secondary antibodies used were fluorescent conjugated secondary antibodies IRDye 680 donkey anti-goat ((LI-COR Biosciences, Cat# 926-68074, RRID:AB_10956736) and IRDye 800 goat anti-rabbit ((LI-COR Biosciences, Cat# 926-32211, RRID:AB_621843). |
| Validation | All antibodies have been used according to manufacturer's instructions. For details of verification , relevant citations or further information see the manufacturer's websites. |

## Animals and other organisms

Policy information about studies involving animals; ARRIVE guidelines recommended for reporting animal research

| | |
|---|---|
| Laboratory animals | This study involved mice. All strains are described in the material and methods section, both female and male mice were involved. The age of the mice in an experiment is either stated in the methods section and/or in the figure/ figure legend for each strain and experiment. |
| Wild animals | This study did not involve wild animals. |
| Field-collected samples | This study did not involve samples collected from the field. |
| Ethics oversight | All animal studies were conducted in accordance with the “Australian code for the care and use of animals for scientific purposes” and were approved by the Animal Ethics Committee of Austin Health. |

Note that full information on the approval of the study protocol must also be provided in the manuscript.
